# Inflammatory biomarkers in pregnant women with COVID-19: a retrospective cohort study

**DOI:** 10.1038/s41598-021-92885-7

**Published:** 2021-06-25

**Authors:** Andrea Lombardi, Silvia Duiella, Letizia Li Piani, Agnese Comelli, Ferruccio Ceriotti, Massimo Oggioni, Antonio Muscatello, Alessandra Bandera, Andrea Gori, Enrico Ferrazzi

**Affiliations:** 1 Infectious Diseases Unit, Foundation IRCCS Ca’ Granda Ospedale Maggiore Policlinico, Via Francesco Sforza 35, 20122 Milan, Italy; 2 Unit of Obstetric, Dept of Woman Child and Neonate, Foundation IRCCS Ca’ Granda Ospedale Maggiore Policlinico, Mangiagalli Centre, University of Milan, Milan, Italy; 3 Clinical Laboratory, Foundation IRCCS Ca’ Granda Ospedale Maggiore Policlinico, Milan, Italy; 4 Department of Pathophysiology and Transplantation, University of Milan, Milan, Italy

**Keywords:** Predictive markers, Viral infection

## Abstract

Coronavirus disease 2019 (COVID-19) is a pandemic viral disease affecting also obstetric patients and uncertainties exist about the prognostic role of inflammatory biomarkers and hemocytometry values in patients with this infection. To clarify that, we have assessed the values of several inflammatory biomarkers and hemocytometry variables in a cohort of obstetric patients hospitalized with COVID-19 and we have correlated the values at admission with the need of oxygen supplementation during the hospitalization. Overall, among 62 (27.3%) pregnant women and 165 (72.7%) postpartum women, 21 (9.2%) patients received oxygen supplementation and 2 (0.9%) required admission to intensive care unit but none died. During hospitalization leukocytes (p < 0.001), neutrophils (p < 0.001), neutrophils to lymphocytes ratio (p < 0.001) and C reactive protein (p < 0.001) decreased significantly, whereas lymphocytes (p < 0.001), platelets (p < 0.001) and ferritin (p = 0.001) increased. Lymphocyte values at admission were correlated with oxygen need, with a 26% higher risk of oxygen supplementation for each 1000 cells decreases. Overall, in obstetric patients hospitalized with COVID-19, C reactive protein is the inflammatory biomarker that better mirrors the course of the disease whereas D-dimer or ferritin are not reliable predictors of poor outcome. Care to the need of oxygen supplementation should be reserved to patients with reduced lymphocyte values at admission.

## Introduction

The pandemic of coronavirus disease 2019 (COVID-2019), caused by the severe acute respiratory syndrome coronavirus 2 (SARS-CoV-2), is affecting many women during pregnancy and in the postpartum worldwide. Even if the earliest data from China indicated that pregnant women infected by SARS-CoV-2 did not experience a worse outcome than non-pregnant women of the same age^1^, according to newest reports, pregnant women are more likely to deliver preterm, to have an increased risk of maternal death and of being admitted to the intensive care unit (ICU)^2,3^. In addition to its impact on the cardio-pulmonary physiology, pregnancy is characterized by several shifts in the woman immunologic profile. Particularly, in preparation of delivery, a pro-inflammatory state occurs with immune cells migrating into the myometrium and high levels of pro-inflammatory cytokines found both in the cervical tissue and in the peripheral blood^4^. Unfortunately, a peculiar characteristic of COVID-19 is the release of a large amount of inflammatory cytokines, a condition that in severe cases resembles the macrophages activation syndrome^5^. Some inflammatory biomarkers have been considered as tools to monitor the evolution of COVID-19, namely C reactive protein (CRP), lactate dehydrogenase (LDH), ferritin and D-dimer^6–8^. Also alterations of the leukocytes count, such as lymphocytopenia or an elevated neutrophils to lymphocytes ratio (NLR), seems correlated with disease severity^9,10^.

Unfortunately, the role of the above-mentioned markers has not been studied during pregnancy and in the postpartum in women infected by SARS-CoV-2. We do not know their evolution during the disease and whether their values can be adopted at admission to guide clinical choices. In this retrospective cohort of obstetric patients admitted with COVID-19 at our tertiary referral centre, we assessed the baseline values and the trend of inflammatory biomarkers and hemocytometry variables and their association with the severity of COVID-19.

## Results

### Demographic and clinical characteristics

We enrolled 227 women. At discharge, 62 patients (27.3%) were still pregnant, whereas 165 (72.7%) delivered during the hospitalization. The median gestational age at admission was 34 weeks and 3 days. Overall, no death occurred in our cohort, 21 (9.2%) patients required oxygen supplementation, and only 2 (0.9%) were admitted to the ICU. In both cases the reason was an acute pulmonary embolism leading to respiratory insufficiency in patients not receiving venous thromboembolism prophylaxis with enoxaparin. Pregnant women had higher mean body mass index (p < 0.05) and lower gestational age (p < 0.05) compared with those who delivered and reached the virologic cure in a longer period (p < 0.05) and reported more frequently fever, dyspnoea, cough and asthenia as symptoms (p < 0.05). Since March 30, 2020, all patient (200, 87.7%) started at admission prophylactic enoxaparin at the dosage of 100 IU/Kg daily. Immunomodulatory therapy with hydroxychloroquine 200 mg every 12 h was started until May 2020 in those with lung involvement at chest X-ray (20, 8.8%). Table 1 shows the clinical and demographic characteristics of the patients enrolled.

**Table 1:** Demographic and clinical characteristics of obstetric patients with COVID-19.

	All (n = 227)	Postpartum women (n = 165)	Pregnant women (n = 62)	p
Age (years)	31.9 (6.1)	32 (6.3)	31 (5.8)	
**Ethnicity**				
Caucasic	125 (54.8%)	100 (60.6%)	25 (40.3%)	< 0.05
Black	4 (1.8%)	1 (0.6%)	3 (4.8%)	< 0.05
Hispanic	50 (21.9%)	31 (18.8%)	19 (30.6%)	< 0.05
Arab	48 (21.1%)	33 (20%)	15 (24.2%)	ns
BMI	24.6 (5.4)	23.8 (5.7)	28.1 (5)	< 0.05
Gestational age at admission (weeks + days)	34 + 3	37 + 2	27.4 + 5	< 0.05
Time since symptoms (days)	4.7 (3.7)	4.6 (3.84)	5.4 (2)	ns
Hospitalization length (days)	10.5 (8.9)	10.9 (9.8)	9.1(6.2)	ns
Time for clinical cure (days)	6.4 (5.2)	5.1 (3.7)	8.3 (6.5)	< 0.05
Time for virologic cure (days)	22 (33.8)	22.5 (42)	22 (9.8)	ns
**Symptoms**	102 (54.8%)	50 (30.3%)	52 (83.8%)	< 0.05
Fever	60 (26.3%)	36 (21.8%)	24 (38.7%)	< 0.05
Dyspnoea	28 (12.3%)	9 (5.4%)	19 (30.6%)	< 0.05
Cough	64 (28.1%)	25 (15.1%)	39 (62.9%)	< 0.05
Coryza	35 (15.4%)	22 (13.3%)	13 (21%)	ns
Asthenia	22 (9.6%)	5 (3%)	17 (27.4%)	< 0.05
Anosmia/ageusia	13 (5.7%)	7 (4.2%)	6 (9.7%)	< 0.05
Nausea/diarrhoea	7 (3.1%)	4 (2.42%)	3 (4.8%)	ns
Pharyngitis	22 (9.6%)	13 (7.9%)	9 (14.5%)	ns
**Chest X-ray/Chest computed tomography**	88 (38.6%)	42 (25.4%)	46 (74.2%)	
Reduced transparency/Ground glass	35 (15.4%)	12 (7.3%)	23 (37.1%)	< 0.05
Interstitial thickening/Crazy paving	45 (19.7%)	19 (11.5%)	26 (42%)	< 0.05
Consolidation	25 (11%)	11 (6.6%)	14 (22.6%)	ns
**Therapy**				
Lopinavir/Ritonavir	1 (0.4%)	0	1 (2.1%)	ns
Hydroxychloroquine	20 (8.8%)	13 (7.9%)	7 (11.3%)	ns
LMWH	200 (87.7%)	151 (95.5%)	49 (79%)	< 0.05
Remdesivir	1 (0.4%)	0	1 (2.1%)	ns
Oxygen supplementation	21 (9.2%)	8 (4.8%)	13 (21%)	< 0.05

Among pregnant women are included those patients who did not deliver during hospitalization, among postpartum women those who did. All data are expressed as mean with standard deviation. (*BMI* body mass index, *LMWH* low-molecular-weight heparin, *CPAP* continuous positive airways pressure, *ICU* intensive care unit).

### Inflammatory biomarkers and white blood cells trend during hospitalization

**Figure 1:**
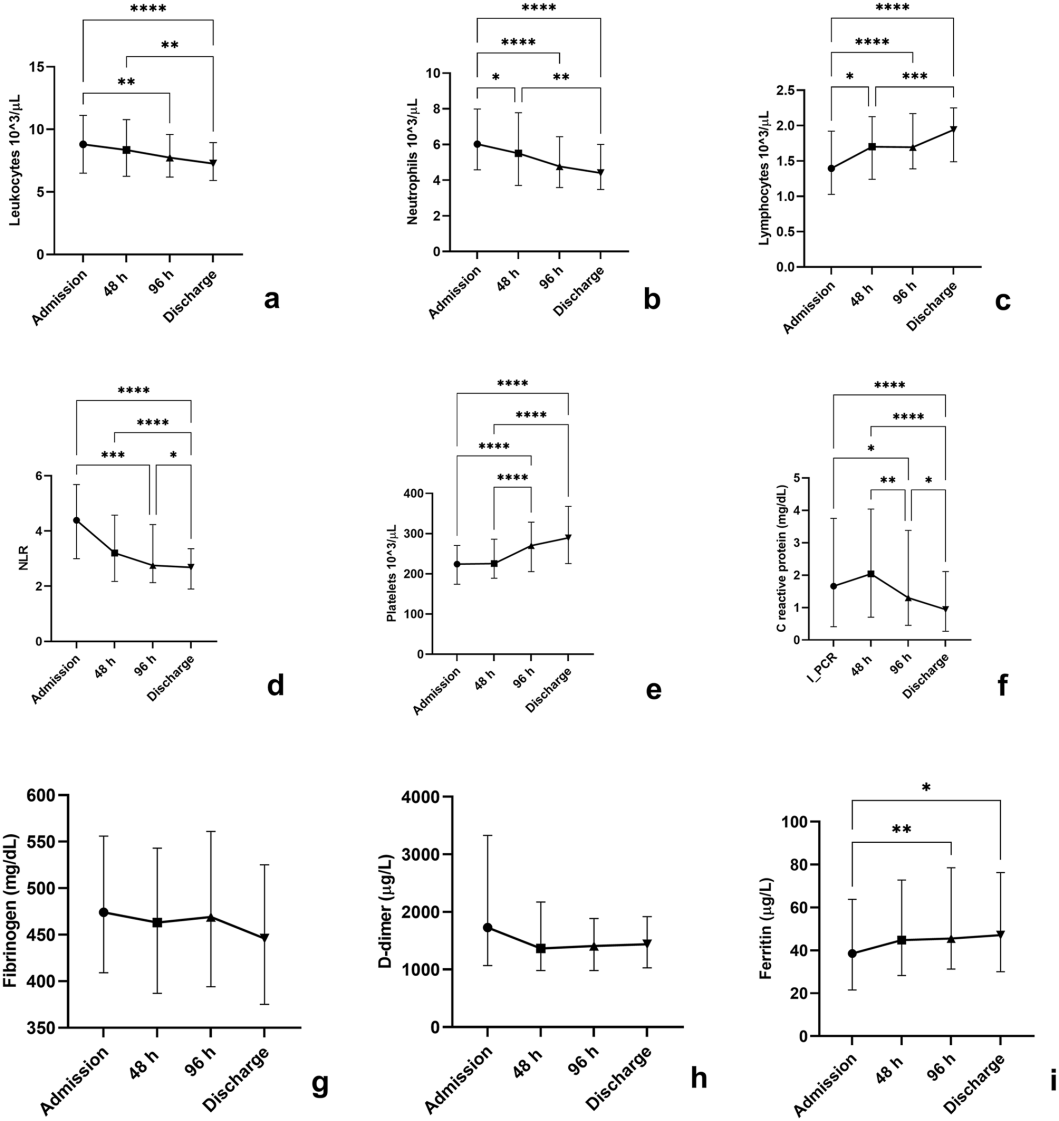
Leukocytes (**a**), neutrophils (**b**), lymphocytes (**c**), neutrophils to lymphocytes (NLR) ratio (**d**), platelets (**e**), C reactive protein (**f**), fibrinogen (**g**), D-dimer (**h**) and ferritin (**i**) values assessed at admission, at 48 and 96 h from admission and at discharge. Data are shown as mean with standard deviation. *P ≤ 0.05, **P ≤ 0.01, ***P ≤ 0.001, ****P ≤ 0.0001.

At admission, the median lymphocytes value was 1,395 cells/µL. The median values of NLR, C reactive protein, D-dimer, procalcitonin and fibrinogen were all above the upper reference limits (4.4, 1.66 mg/dL, 1,727 µg/L, 0.06 µg/L and 474 mg/dL, respectively). During the observation time leukocytes (p < 0.001), neutrophils (p < 0.001), NLR (p < 0.001) and CRP (p < 0.001) values decreased whereas lymphocytes (p < 0.001), platelets (p < 0.001) and ferritin (p = 0.001) increased. Table 2 summarizes inflammatory biomarkers and white blood cells trend during hospitalization. The results of multiple comparisons tests performed to assess differences among the same variable at different timepoints are shown in Fig. 1.

**Table 2:** Values (median and interquartile range) of inflammatory biomarkers and hemocytometry variables at different study timepoints.

	Admission	48 h	96 h	Discharge	p
Leukocytes (cells/mL)	8810 (6510–11,120)	8360 (6255–10,790)	7740 (6190–9595)	7270 (5920–8940)	< 0.001
Neutrophils (cells/mL)	6020 (4580–7990)	5500 (3700–7780)	4770 (3590–6440)	4400 (3480–6000)	< 0.001
Lymphocytes (cells/mL)	1395 (1028–1923)	1700 (1240–2125)	1695 (1388–2170)	1940 (1488–2250)	< 0.001
Platelets (10^6^ cells/mL)	224 (174–270)	225 (189–286)	270 (205–328)	289 (225–367)	< 0.001
NLR	4.4 (3–5.7)	3.2 (2.2–4.6)	2.7 (2.1–4.2)	2.7 (1.9–3.3)	< 0.001
Ferritin (µg/L)	38.5 (21.5–64)	44.8 (28.2–72.7)	45.5 (31.2–78.5))	47.1 (30–76.2)	< 0.001
C reactive protein (mg/dL)	1.66 (0.41–3.75)	2 (0.7–4)	1.3 (0.4–3.4)	0.9 (0.3–2.1)	< 0.001
D-dimer (µg/L)	1727 (1069–3328)	1364 (980–2,171)	1408 (982–1886)	1439 (1029–1917)	0.0248
Procalcitonin (µg/L)	0.06 (0.045–0.1)	0.06 (0.04–0.08)	0.05 (0.03–0.07)	0.04 (0.03–0.06)	< 0.001
Fibrinogen (mg/dL)	474 (409–556)	463 (387–543)	469 (394–561)	446 (375–525)	ns
LDH (U/L)	186 (155–236)	189 (141–245)	203 (154–264)	166 (138–214)	ns
AST (U/L)	59 (41–107)	55 (45–82)	53 (42–72)	45 (36–54)	0.042
ALT (U/L)	17 (13–24)	19 (13–29)	24 (15–40)	24 (15–40)	< 0.001

*NLR* neutrophils to lymphocytes ratio, *LDH* lactate dehydrogenase, *AST* aspartate aminotransferase, *ALT* alanine aminotransferase.

### Predictors of oxygen need

The independent effect of different variables at baseline on the likelihood of receiving oxygen supplementation was interrogated by a univariate logistic regression analysis and the results are reported in Table 3. The model proved to be significant only for lymphocytes and explained 94% (Nagelkerke R^2^) of the variance. Reduced lymphocytes value was associated with an increased likelihood of oxygen supplementation. A multiple logistic regression model was built, including lymphocytes, CRP and D-dimer. Lymphocytes value maintained its association with oxygen supplementation in the multivariate model.

**Table 3:** Simple logistic regression and multiple logistic regression models for oxygen supplementation at baseline. In the multivariate logistic regression were included lymphocytes, CRP and D-dimer.

	Simple logistic regression		Multiple logistic regression	
	Odds ratio (95%CI)	p	Odds ratio (95%CI)	p
Leukocytes	0.94 (0.82–1.05)	ns		
Neutrophils	1.021 (0.88–1.15)	ns		
Lymphocytes	0.26 (0.10–0.7)	0.005	4.5 (1.34–15.1)	0.02
NLR	1.108 (0.977–1.246)	ns		
CRP	1.008 (0.958–1.036)	ns	1 (0.5–1.02)	ns
PCT	1.90 (0.024–19.20)	ns		
Fibrinogen	1.001 (0.996–1.005)	ns		
d-Dimer	1 (0.99–1)	ns	1 (1–1)	ns
Haemoglobin	0.77 (0.54–1.01)	ns		
Ferritin	1.003 (0.99–1.01)	ns		

*NLR* neutrophils to lymphocytes ratio, *CRP* C reactive protein, *PCT* procalcitonin, *CI* confidence interval.

## Discussion

Our findings show how, among the variables assessed in our study, CRP was the inflammatory biomarker which varied more significantly during the course of COVID-19 in obstetric patients, supporting its employment as a tool to monitor the evolution of the disease. Among hemocytometry parameters, leukocytes, neutrophils, lymphocytes, platelets, and NLR are those with values which changed significantly. Considering their widespread availability in any hospitalized patient, they also should be considered in assessing disease progression. Interestingly, low lymphocytes value at admission was associated with a higher likelihood of receiving oxygen supplementation during hospitalization, suggesting that additional care should be reserved to patients presenting with reduced values of these blood cells.

Our findings are in agreement with the results of previous studies highlighting how COVID-19 patients are characterized by a relevant systemic inflammation, which acts as a driver of morbidity and mortality. Particularly, lymphocytopenia^9^, a high NLR^11^ and high values of CRP^12^ had all been linked with disease severity or mortality. Our work confirms that these variables show abnormal values also in obstetric patients and mirrors the course of the disease.

Intriguingly, D-dimer values, despite being well above the upper reference limit, were not associated with oxygen need and did not vary significantly during the hospitalization. A mounting amount of evidence is supporting the role of vascular endothelialitis and thrombosis in the pathogenesis of COVID-19^13,14^, and D-dimer values above 1000 µg/L have been associated with a poor prognosis^15^. In our cohort D-dimer values were above this limit in all the timepoints. It is possible that the administration of antithrombotic prophylaxis with enoxaparin in almost 90% of the patients have prevented some additional thromboembolic events. Moreover, it should be recognized that pregnancy is characterized by a physiologic increase of D-dimer values, especially in the third trimester^16,17^. However, in the specific setting of pregnant women, published data on the prognostic role of D-dimer are so far conflicting and further study are needed^18,19^.

To our surprise ferritin values were well above those observed in normal pregnancies^20^, which is characterized by a physiologic anaemic state and where high levels of ferritin, especially in the third trimester, are associated with negative outcomes (e.g. preterm delivery and gestational diabetes)^21^. At the same time, our values were well below those observed in several cohorts of non-pregnant COVID-19 patients^22^. For example, Chen and colleagues observed a median ferritin of 337 µg/L among their patients with moderate COVID-19^8^. Therefore, it is possible to speculate that the inflammatory status caused by SARS-CoV-2 leads to an increase of serum ferritin also in obstetric patients, but this increase is partially concealed by the low levels of this molecule physiologically encountered in this population.

When compared to similar cohorts of obstetric patients with COVID-19, we have a striking similarity in terms of displayed symptoms. Fever, cough and dyspnoea were the three most frequently reported symptoms also in the large British UKOSS (UK Obstetric Surveillance System) cohort and in two Italian cohorts^23–25^. Interestingly, we have found that the severity of COVID-19 in pregnant women or in the postpartum was inferior to that previously reported in the literature. Indeed, we did not observe any death and admission to the ICU was required in only two patients. Di Toro and colleagues in their systematic review of the literature reported an admission rate to the ICU of 8%, with 3 stillbirths and 5 maternal deaths^26^.

Several differences emerged between the two groups of obstetric patients composing our cohort, those who did not deliver during hospitalization (pregnant women) and those who did (postpartum women). The first group displayed a more severe clinical pattern, with a longer time to clinical cure, a higher prevalence of symptoms, more radiographic findings, and a higher need of oxygen. Our study was not designed to investigate the causes of this difference, but it could be speculated that the presence of the foetus, compressing the other abdominal viscera and reducing the space for lung expansion, could have exacerbated symptoms such as dyspnoea and justify the higher need of oxygen in this group. This physical condition hardly explains the higher frequency of fever reported among pregnant women, which could be related to the different hormonal asset specific of this group. It is a well-known fact that during gestation pregnancy-associated hormones alter the immune response and disease pathogenesis, with other respiratory viral infection such as influenza occurring with enhanced severity in this population^27^. Unfortunately, quantification of hormones was not performed in our cohort, but these findings suggest that efforts should be devoted in understanding the impact of sexual hormones on COVID-19 severity.

We recognize that our work has some limitations. First, is a single centre study, and therefore the conclusions can apply only to the specific population afferent to the hospital in which the study was performed. Considering that our hospital was the referring centre for obstetric patients with COVID-19 of a large portion of Lombardy region, the most populous Italian region, and the large variation in ethnicities taken in care, we can suggest that this element did not impact the results. Second, it is a retrospective study, and some analysis (i.d. surveillance nasal swab) were performed for clinical/epidemiologic reasons and not to ascertain biologic differences.

Based on the results of our study, a minimum set of analyses composed of hemocytometry plus CRP should be performed in obstetric patients with COVID-19 at admission and during hospitalization, to assess disease severity and follow the evolution of the disease. Specific care to oxygen need should be reserved to those patients presenting with low values of leukocytes and lymphocytes at admission.

## Methods

### Study population

We enrolled a cohort of pregnant women consecutively admitted at the COVID-19 Maternity Hub at the Foundation IRCCS Ca' Granda Ospedale Maggiore Policlinico in Milan, Italy with a diagnosis of COVID-19 in the period from March 10, 2020 to April 24, 2020. Clinical and demographic data were extracted from the electronic medical records.

The diagnosis of COVID-19 was established by the detection of SARS-CoV-2 in a nasopharyngeal swab. The nasopharyngeal swab was performed either in all pregnant women admitted for obstetric indication or in women with at least one of the following signs or symptoms: fever, cough, dyspnoea, asthenia, myalgia, coryza, sore throat, headache, ageusia or dysgeusia, anosmia or parosmia, ocular symptoms, diarrhoea, nausea, and vomit. All SARS-CoV-2 positive cases were admitted in a dedicated COVID-19 ward. Inflammatory biomarkers and hemocytometry variables were assessed at admission, at 48 h and 96 h after admission and at discharge. The execution of chest X-ray or chest computed tomography (CT) was reserved to patients with respiratory symptoms, according to clinical judgment. The patients enrolled were subdivided in two groups: women who delivered during hospitalization or women who did not.

Clinical cure was defined as the absence of fever, a respiratory rate < 22 breaths per minutes and peripheral haemoglobin saturation values > 95% for at least three days. All patients repeated two nasopharyngeal swabs at an interval of 24 h, 14 days after discharge or 14 days after clinical cure if still hospitalized for other reasons until October 2020. If both the swabs resulted negative the patient was considered cured (virologic cure) of the infection and not requiring anymore isolation measures anymore. Since October 2020 only one nasopharyngeal swab to assess virologic cure was performed, 10 days after the first positive nasopharyngeal swab or 10 days after symptoms onset.

### Pro-inflammatory biomarkers assessment and white blood cell profile

Procalcitonin (PCT) and ferritin were measured with electrochemiluminescent immunoassays (ECLIA) on a Roche Cobas e801 instrument (Roche Diagnostics, Monza, Italy). C-reactive protein (CRP) was measured with an immunoturbidimetric method and lactate dehydrogenase (LDH), alanine and aspartate aminotransferase (ALT, AST) with the International Federation of Clinical Chemistry (IFCC) optimized methods on a Roche Cobas c702 instrument (Roche Diagnostics, Monza, Italy). Fibrinogen and D-dimer were measured with ACL Top (Werfen, Milano, Italy). Hemocytometry analysis was performed with Sysmex XN 9000 (Dasit, Cornaredo, Italy).

### SARS-CoV-2 detection

Two different methods were used for viral detection. The first one consisted in Seegene Inc reagents (Seoul, Korea), RNA extraction with STARMag Universal Universal Cartridge kit on Nimbus instrument (Hamilton, Agrate Brianza, Italy) and amplification with Allplex 2019-nCoV assay, while the second employed a GeneFinder COVID-19 Plus RealAmp Kit (OSANG Healthcare, Anyangcheondong-ro, Dongan-gu, Anyang-si, Gyeonggi-do, Korea) on ELITech InGenius instrument (Torino, Italy). Both assays identify the virus by multiplex rRT-PCR targeting three viral genes (E, RdRP and N).

### Statistical analysis and ethics

Descriptive statistics were obtained for all the variables reported for this study. Groups were compared with unpaired t test or Mann–Whitney test and Fisher's exact test. The trend of different variables during hospitalization was assessed with Friedmann test and Dunn's multiple comparisons test. Univariate logistic regression models were employed to assess correlation between variables at baseline and oxygen supplementation. Multivariate logistic regression models were then built including significant variables at univariate analysis and variables with a biologic correlate. A *p* value < 0.05 was deemed statistically significant. All the analysis was performed with SPSS Statistics 23 (IBM Corp, USA). The study was approved by the Comitato Etico Milano Area 2 (#339_2020) and conducted according to the Declaration of Helsinki. An informed consent was obtained from all the patients enrolled.

## Data Availability

All data will be available on request.
